# Aberrant pulmonary immune response of obese mice to periodontal infection

**DOI:** 10.1515/biol-2022-0089

**Published:** 2022-08-17

**Authors:** Wei Zhou, Dongying Xuan, Ting Yu, Jincai Zhang

**Affiliations:** Department of Periodontics, Shenzhen Stomatological Hospital, Southern Medical University, Shenzhen, Guangdong, China; Southern Medical University, No. 1023-1063, Shatai South Road, Baiyun District, Guangzhou, Guangdong, China; Department of Periodontics, Affiliated Stomatology Hospital of Guangzhou Medical University, Guangdong Engineering Research Center of Oral Restoration and Reconstruction, Guangzhou Key laboratory of Basic and Applied Research of Oral Regenerative Medicine, 195A, Dongfeng West Road, Yuexiu District, Guangzhou, Guangdong, China

**Keywords:** pulmonary immune response, periodontal infection, diet-induced obesity

## Abstract

Obesity and periodontitis constitute mutual risk factors in respiratory disorders; this study aimed to explore the pulmonary immune response to periodontal infection using combined animal models with diet-induced obesity (DIO). Thirty-two C57 BL/6J mice were randomly divided into low-fat (LF) or high-fat (HF) diet groups and fed an LF diet as a control or an HF diet to induce obesity. The 30-week mice in the diet group were divided into periodontal ligation group (10 days using *Porphyromonas gingivalis* ATCC 33277) or sham-ligation group. The expressions of the macrophage-specific maker (F4/80), macrophage chemotactic protein1 (MCP1), and inflammatory cytokines in lung tissues were analyzed. The mRNA and protein levels of F4/80, MCP1, interleukin (IL)-1β, and IL-6 expressions were significantly upregulated by obesity in lung tissues. However, the mRNA and protein levels of F4/80, MCP1, and IL-6 were downregulated by periodontitis in DIO mice relative to that of the HF control group. Periodontitis increased tumor necrosis factor-α level of lung tissues under LF, while IL-10 was not affected by obesity regardless of periodontitis. Periodontitis may aggravate pulmonary immune response in obese rodents. This may relate to the imbalance of the pro- and anti-inflammatory cytokine status of lung lesions, which tends to attenuate the infiltration of alveolar macrophages.

## 1 Introduction

Periodontitis is a nonspecific, highly prevalent dysbiosis-related chronic infection characterized by the progressive destruction of periodontal supporting tissues, which can cause systemic inflammation and infectious metastasis with potential impact on distant organs [1]. It ranks second among the most prevalent oral diseases worldwide and has become a significant public health concern [2]. Previous reports have revealed the relationship between periodontitis and top-ten causes of death, such as diabetes, COVID-19 infection, cardiovascular diseases, respiratory disease [3,4,5], and even premature mortality [6]. Extensive clinical studies clearly show that periodontitis may increase the risk of various pneumonia diseases, including chronic obstructive pulmonary disease (COPD), pneumonia, and asthma [7,8]. The anatomical continuity between the lungs and the oral cavity allows dental plaque to affect lung flora [9]. Some anaerobic periodontal pathogens, especially *Porphyromonas gingivalis*, are common isolates derived from the lungs of patients with infectious pneumonia and COPD [10]. In animal studies, the intratracheal challenge with *P. gingivalis* is responsible for persistent inflammatory responses in the lungs, which involves cell recruitment and proinflammatory cytokine production [11,12]. Extensive endeavors have either focused on epidemiological data or used intratracheal challenges with periodontal pathogens in an animal model to elucidate the relationship between periodontitis and respiratory disease. Recent studies showed that *P. gingivalis* was detected in the gingival, tongue, and lung tissues after 6 weeks of oral inoculation, implying that the systemic immunity induced by periodontitis can alter immune response at distant sites only after a longer period [13,14].

From the immunology viewpoint, the immune response of lung tissue is largely dependent on the nutritional status of the organism; specifically, malnutrition or over-nutrition will accordingly give rise to immune suppression or dysfunction. Periodontitis and respiratory disorders are likely to raise multiple risk factors, such as obesity [15]. It is well documented that obesity has profound effects on asthma, acute respiratory distress syndrome, obstructive sleep apnea, and lung infection (as witnessed in COVID-19, H1N1 pandemic, and else) [16,17]. Obesity alters the mechanical properties of the respiratory system. Furthermore, it is a chronic low-grade metaflammation with many immunometabolic dysregulations, such as systemic inflammation, dyslipidemia, hyperglycemia, and insulin resistance [18]. Such obesity-derived systemic alterations weaken host immunity or demonstrate an overexuberant inflammatory response, which finally increases the susceptibility of the lung to injury. Diet-induced obesity (DIO) was shown to exacerbate lung inflammation through enhanced eosinophil trafficking from bone marrow to lung tissues in a murine model of allergic asthma [19] and reduced neutrophil recruitment upon exposure to ozone [20]. The combined effects resulted in a disturbance of the alveolar-capillary barrier and led to an increased susceptibility to particle-induced lung inflammation [21]. These findings indicate that obesity, as a special state of the body, may exacerbate lung changes when subjected to other stimuli.

Impaired host response has been considered a common mechanism linking obesity with periodontitis. When established in individuals with obesity, periodontitis is then prone to intensify the systemic inflammatory state triggered by the proinflammatory cytokines and dissemination of bacterial products [15]. Periodontitis mice induced by *P. gingivalis* coupled with ligature over a long period can afford pulmonary inflammation, in which cytokines play an important role in the lung [14]. Nevertheless, the effect of periodontitis on the occurrence and development of pulmonary immune response in the presence of obesity remains largely unexplored. We hypothesized that the influence of periodontitis on the onset and progression of respiratory disease might be amplified or accelerated in obese cases. Therefore, this study aimed to explore the pulmonary immune response to periodontal infection in the context of DIO using combined animal models.

## 2 Materials and methods

### 2.1 Animals

Animals were obtained from and cultured in Guangdong Medical Laboratory Animal Center, China. Thirty-two C57 BL/6J mice (male, 6 weeks old) were randomly divided into two groups: low-fat (LF/LF^−^) and high-fat (HF/HF^−^) diet groups (*n* = 16 per group). The HF group was fed a 60% kcal HF diet for 30 weeks to induce obesity (DIO), while the LF group was fed a 10% kcal LF diet (D12492 and D2450B, Research Diet Inc., NJ, USA) as the normal-weight control. All the mice were group-housed (3–5 mice per cage) *ad libitum* in a specific pathogen-free environment, followed by a 10–14-h day-to-night cycle, with free access to diet and water. Body weight was measured every 2 weeks. Sixteen-hour (overnight) fasting blood glucose was measured by a glucometer at 0 and 30 weeks. After 30 weeks, the two diet groups were divided into periodontitis (−P) and periodontal health control (−C) groups (*n* = 8) based on the body-weight matching rule. *P. gingivalis* ATCC33277-lyophilized powder (ATCC, Manassas, VA, USA) was suspended in sterilized water, evenly coated in an anaerobic blood plate, and cultured in an anaerobic chamber for 7 days (90% N_2_, 5% CO_2_, 5% H_2_ at 37°C). Subsequently, the smallest colony was selected and cultured in an 8 mL anaerobic broth in the same anaerobic environment for 1–2 days to afford turbid broth, which was then used as a working broth [22].

Under anesthesia with 4% w/v chloral hydrate (i.p.), the P group was ligated bilaterally at maxillary second molars using a 5–0 silk ligature presoaked in the working broth for 24 h. The C group was sham-ligated at the same sites with sterile silk, which was then removed immediately. On day 10, all the mice were euthanized by cardiac puncture [23]. Visceral adipose tissues, including perirenal white adipose tissue (PWAT), mesenteric white adipose tissue (MWAT), and epididymal white adipose tissue (EWAT), were resected and weighed. The bilateral maxilla and lungs were removed and dissected. For the morphometric analysis, one side of the alveolar bone was fixed in 4% w/v paraformaldehyde. The inferior lobe of the left lung was fixed in 4% formaldehyde for histopathological examination. The other lungs were snap-frozen in liquid nitrogen for RNA extraction.


**Ethical approval:** The research related to animal use has been complied with all the relevant national regulations and institutional policies for the care and use of animals. The Animal Experimental Committee of Southern Medical University approved all experimental protocols. The study protocol followed all recommendations of the National Institutes of Health Guide for the care and use of laboratory animals [24].

### 2.2 Morphometrical analysis

The bone tissue was fleshed and stained with 1% methylene blue (MP Biomedicals, Shanghai, China) according to the reported method [25]. The frontal view of the stained jaw was captured using the stereomicroscope (magnification ×30). Distances from the alveolar bone crest (ABC) to the cemental enamel junction (CEJ) at 18 sites of the three molars were measured and averaged as vertical bone loss (VBL) [23].

### 2.3 Histology

Fixed lung samples were dehydrated in graded alcohol series, cleared with dimethylbenzene, and embedded in paraffin. Serial sections (thickness: 4 mm) were stained with hematoxylin–eosin (H&E) for immunohistochemistry analyses.

Immunohistochemistry was carried out in two steps. After deparaffinization and hydration, the lung sections were boiled in EDTA solution (pH 9.0) for 15 min in a microwave.

Incubation with rabbit anti-mouse primary antibodies F4/80 (markers for macrophages) (1:500, GB11027; Servicebio), macrophage chemotactic protein (*MCP1*) (1:500, GB11199; Servicebio), tumor necrosis factor (*TNF*)*-α* (1:300, GB11188; Servicebio), interleukin (*IL*)*-1β* (1:800, GB11113; Servicebio), *IL-6* (1:800, GB11117; Servicebio), and *IL-10* (1:300, GB11108; Servicebio) was performed overnight at 4°C. Incubation with a ready-to-use secondary antibody coupled with horseradish peroxidase (goat-anti-rabbit) (1:200, GB23303; Servicebio) was carried out and continued for 40 min at 37°C, and 3,3′-diaminobenzidine (G1211; Servicebio) was used for the color reaction. A blank control was incubated with phosphate-buffered saline instead of a primary antibody. Each section with four fields, including two fields of peri-bronchiolar areas and two fields of the alveolar septum, was analyzed by a microscope with a camera system (XSP-C204, CIC). To quantify the protein expressions in the specimens, the integrated optical density (IOD) in the area of interest in the pictures was quantified by an analyzing system (Image-Pro^@^ Plus Version 6.0; Media Cybernetics, Inc., Bethesda). The final IOD value was calculated by averaging the total scores of four fields for each section.

### 2.4 RNA isolation and RT-PCR

The total RNA of the lung was extracted using TRIzol reagent (TRIzol, Takara Bio, Kusatsu, Japan) and reversely transcribed (PrimeScript RT reagent kit, Takara Bio, Otsu, Japan) following the manufacturer’s instructions. Primer sequences for the target genes were as follows (5′–3′, forward and reverse):

*MCP1*: ATTTCCACACTTCTATGCCTCCT and ATCCAGTATGGTCCTGAAGATCA;

*TNF-α*: CAACGGCATGGATCTCAAAGAC and CTTGAAGAGAACCTG GGAGTAGAC;

*IL-1β*: GAAATGCCACCTTTTGACAGTG and TGGATGCTCTCATCAGGACAG;

*IL-6*: TCTATACCACTTCACAAGTCGGA and GAATTGCCATTGCACAACTCTTT,

*IL-10*: CGGGAAGACAATAACTGCACCC and CGGTTAGCAGTATGTTGTCCAGC.

Glyceraldehyde-3-phosphate dehydrogenase was used as endogenous control. The reversely transcribed product was quantitatively determined using the SYBR Premix Ex Taq PCR kit (Takara Bio, Otsu, Japan). Relative quantification was performed using a real-time PCR analyzing system (7500; Applied Biosystems, Waltham, MA, USA). The expressions of the genes were determined using the 2^−ΔΔCT^ method.

### 2.5 Statistical analysis

Statistical analysis was performed using commercial software (SPSS 22.0; IBM, Armonk, NY, USA). Independent samples in two groups were tested by independent *t*-test. For factorial design, data were analyzed by the two-way analysis of variance, with diet and ligation being the main effects. All data were expressed as mean ± standard deviations (SDs). Typically, *p* < 0.05 was considered statistically significant.

## 3 Results

### 3.1 Establishment of the DIO model and the periodontitis model

**Figure 1:**
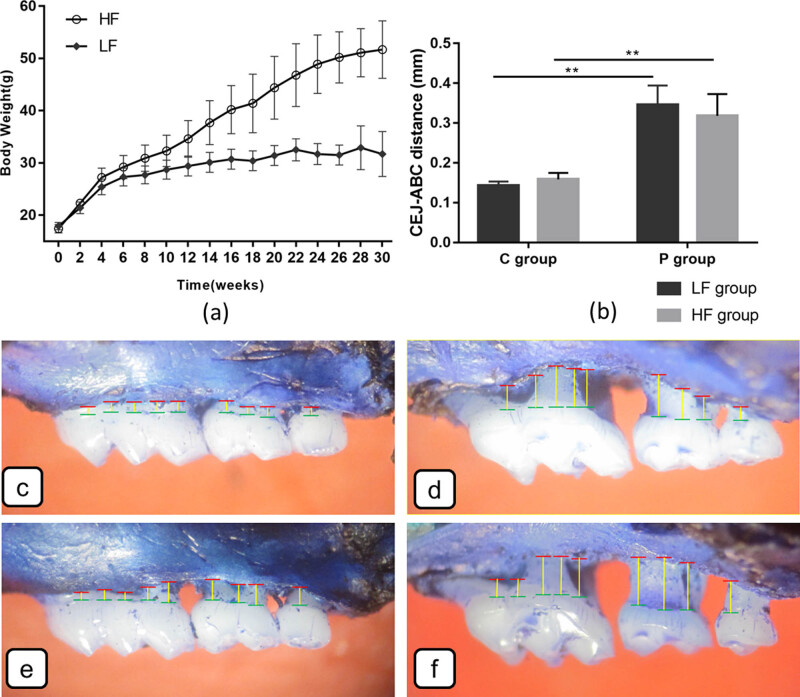
(A) Body weight changes in 6-week-old male C57BL/6 mice fed either with LFD or HFD during the experiment. The measurements were carried out every 2 weeks before 30 weeks and 10 days after ligation (*n* = 8 per group), values are presented as mean ± SD; *ns*, no significance; *p < 0.05; **p < 0.01. (B) The CEJ–ABC distance of periodontitis group (P group) with significantly increased VBL compared with that of the control group (C group) (*n* = 6 per group). (C–F) Representative methylene blue staining of alveolar bone in LFC (C), LFP (D), HFC (E), and HFP (F) groups. Green lines denote ABC level, red lines: CEJ junction level, and yellow lines indicate the distance from CEJ to ABC. Values are presented as mean ± SD**; *ns*, no significance; *p < 0.05; **p < 0.01; black scale bars: 150 μm.

The mice in this study were of uniform weights and had normal blood glucose at the initial stage (Figure 1A). After 30 weeks, the body weight was significantly increased in the HF group (51.70 ± 5.54 g) compared with that of the LF group (31.30 ± 4.26 g) (*p* < 0.001), and so did the body weight after 10 days with sham-ligation/ligation (HF group: 43.2 ± 4.6 g; LF group: 28.7 ± 1.9 g) (*p* < 0.001). Similar tendency was also found in terms of the blood glucose (HF group: 172.23 ± 28.90 mg/dl; LF group: 84.71 ± 22.01 mg/dl) (*p* < 0.001).The weight percentages of white adipose tissues were significantly increased in the HF group than in the LF group (*p* < 0.001), such as EWAT (1.64 ± 0.42 and 0.56 ± 0.26), PWAT (0.87 ± 0.54 and 0.19 ± 0.18), and MWAT (1.05 ± 0.54 and 0.23 ± 0.19).

As clearly shown in Figure 1B, P group had more VBL in periodontium than that of C group (*P* < 0.05). However, VBL was not affected by diet regardless of sham-ligation or ligation.

### 3.2 Recruitment of inflammatory cells in lung tissues with obesity or periodontitis

**Figure 2:**
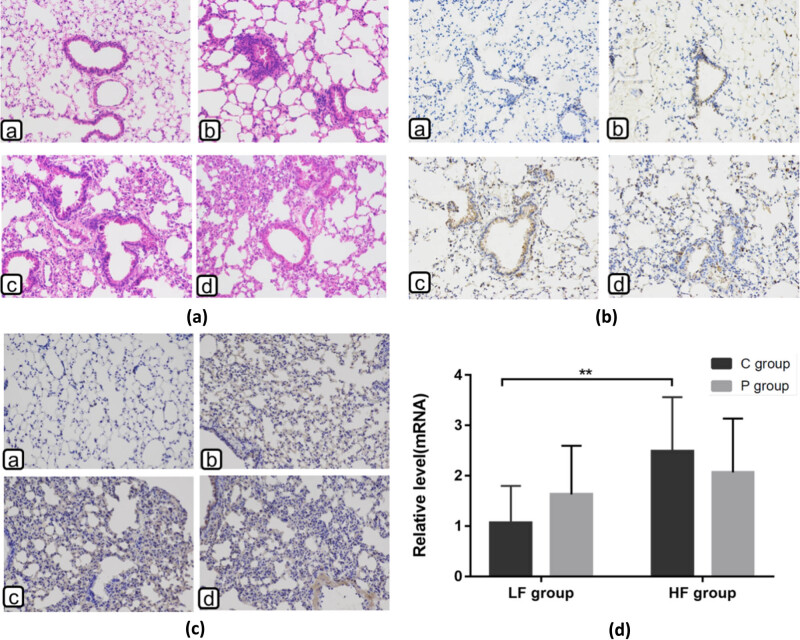
Effects of DIO and periodontitis on lung histopathological changes. (A) Mice lung tissue sections stained with hematoxylin and eosin (H&E) for inflammatory cells in four different groups. (B) The lung tissue sections stained by F4/80 antibody for macrophage infiltration in four different groups. (C) Immunohistochemistry (IHC) staining fields and (D) the mRNA relative expressions of MCP1 (magnification ×200). Groups: LFC (a), LFP (b), HFC (c), or HFP (d). ns, no significance; *p < 0.05; **p < 0.01 (*n* = 8 per group). Black scale bars: 100 μm.

Representative H&E-stained lung sections are shown in Figure 2A to illustrate the differences in pulmonary inflammation among the four groups. LFC mice-featured normal lung architecture (Figure 2A(a)) and the histological features in LFP mice were similar to LFC (Figure 2A(b)), primarily characterized by normal histological features and mild inflammatory cell infiltration. The morphometrical analysis of the lung tissue of HF mice shows alveolar septal thickening and bronchial secretions, which are substantially greater than that of LF mice. In the HF group, mixed infiltrate containing numerous alveolar macrophages and neutrophils around the peribronchiolar region and alveolar spaces are observed (Figure 2A(c) and A(d). Lung inflammation was further defined by increased infiltration of inflammatory cells, which was examined by macrophage maker F4/80 antibody staining (Figure 2B). The majority of infiltrated macrophages was found in peri-bronchiolar regions and alveolar spaces in the HFC and HFP groups (Figure 2B(c) and B(d)). In comparison, the expression of F4/80 macrophages was weaker in the LFC group, which scattered slightly in peri-bronchiolar regions and alveolar spaces in the LFP group (Figure 2B(a) and B(b)). Diet significantly affected F4/80 protein of mice (*F* = 14.975, *p* ≤ 0.01). The HFC group showed an elevated protein level of F4/80 compared with that of the LFC group (HFC vs LFC, *p* ≤ 0.01); however, there was no significant difference between HFP and LFP (*p* > 0.05) or between HF and LF groups (HFP vs HFC, *p* > 0.05; LFP vs LFC, *p* > 0.05).

The protein levels of MCP1 in these groups also showed similar variation behavior as described above. Typically, the diet had a significant effect on MCP1 protein (*F* = 13.433, *p* ≤ 0.01), as manifested by the increased MCP1 level in the HFC group compared to that of the LFC group (HFC vs LFC, *p* ≤ 0.01). The HFP group also showed upregulated protein levels compared to that of the LFP group, without detectable significance (HFP vs LFP, *p* > 0.05). RT-PCR results showed that diet also significantly impacted mRNA level of MCP1 (*F* = 12.78, *p* ≤ 0.01). MCP1 level was doubled in the HFC group than in the LFC group (HFC vs LFC, *p* ≤ 0.01). However, mRNA level of MCP1 was less affected by periodontitis (HFP vs LFP, *p* > 0.05). The LFP group showed upregulated MCP1 mRNA levels without significance compared to that of the LFC group (Figure 2D).

### 3.3 Effect of periodontitis on the expression of inflammatory cytokines in lung tissue

**Figure 3:**
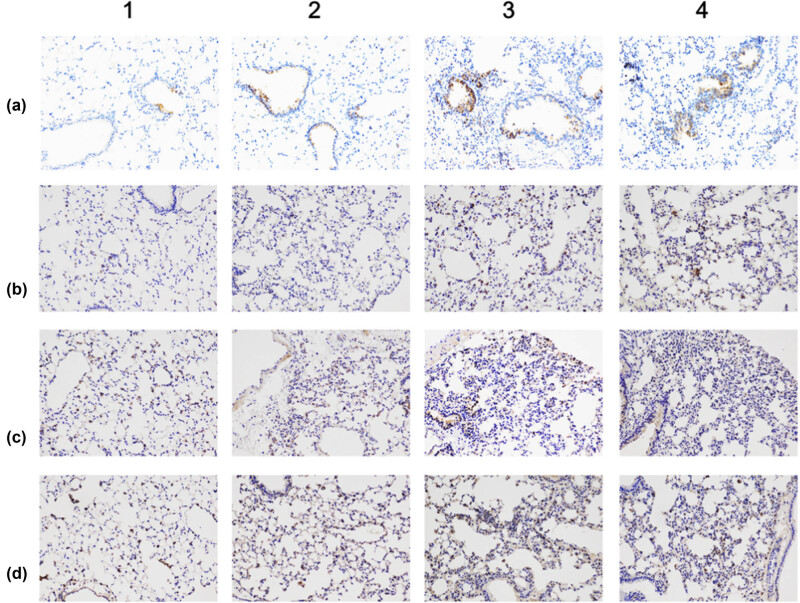
Illustrations of lung inflammation cytokine immunostaining. Columns show the immunohistochemistry staining fields of TNF-α, IL-1β, IL-6, and IL-10 (magnification ×200). Rows show LFC, LFP, HFC, and HFP groups. Black scale bars: 100 μm.

As indicated in Figure 3, immunohistochemistry staining showed that the positive staining areas of TNF-α, IL-1β, and IL-6 proteins were not obvious in LFC; instead, they became more scattered near peri-bronchiolar regions and alveolar spaces in other groups. The immunohistochemistry results showed that diet had a significant effect on TNF-α protein (*F* = 8.41, *p* ≤ 0.01), there was an interaction between the two influencing factors (diet and ligation) that changed TNF-α (*F* = 5.042, *p* ≤ 0.05). Specifically, the LFP group showed increased protein levels of TNF-α (LFP vs LFC, *p* ≤ 0.05). However, the expression decreased without significance in the HF diet groups (HFP vs HFC, *p >* 0.05). The expression of TNF-α in the HFC group was higher than that in the LFC group (HFC vs LFC, *p* ≤ 0.05), but no significant difference was found in the P groups (HFP vs LFP, *p* > 0.05). A similar variation tendency was also found in mRNA levels of TNF-α. The highest expression was found in the HFC group, which was nearly three times higher than that of the LFC group (HFC vs LFC, *p* ≤ 0.05), showing upregulated protein levels when compared with that of the HFP group (HFC vs HFP, *p* > 0.05). The expression of TNF-α in the LFP group was higher than that in the LFC group (LFP vs LFC, *p* ≤ 0.05), but the expression was increased without significance between the HFP and LFP groups (HFP vs LFP, *p* > 0.05).

Regardless of sham-ligation or ligation, the HF group exhibited elevated expression of IL-1β proteins compared to that of the LF group (*p* ≤ 0.01). The relative mRNA expression of IL-1β was also significantly higher in the lung tissues in the context of obesity (HFC vs LFC, HFP vs LFP, *p* ≤ 0.05).

The immunohistochemistry and RT-PCR results showed that diet had a significant effect on IL-6 response level (*p* ≤ 0.05), while the significance was absent in ligation cases (*p* > 0.05). The HFC group showed increased protein level of IL-6 (HFC vs LFC, *p* < 0.05), and the mRNA response was significantly elevated by nearly one-fold in the context of diet (*p* < 0.05). However, the HFP group displayed upregulated IL-6 response without significance compared to that of the LFP group (HFP vs LFP, *p* > 0.05).

**Figure 4:**
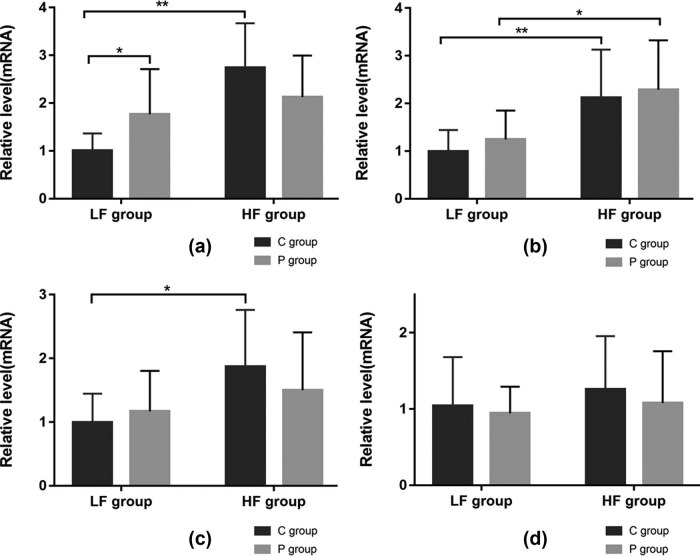
The mRNA relative expressions of TNF-α (A), IL-1β (B), IL-6 (C), and IL-10 (D) in lung tissues of mice. Real-time RT-PCR was performed to calculate the target gene expression in the test sample = 2^−ΔΔCT^. The mRNA level of each group was normalized to the level of LFC and presented as a fold increase. ns, no significance; *p < 0.05; **p < 0.01 (*n* = 8 per group).

The expression of IL-10 proteins was found near alveolar spaces and peri-bronchiolar regions. The results indicate that IL-10 levels were unaffected by either diet or ligation. The differences of TNF-α, IL-1β, IL-6, IL-10 protein, and gene expressions among different groups are shown in Figures 3 and 4.

## 4 Discussion

In this study, we aimed to explore the potential effect of periodontitis on the occurrence and development of lung inflammation status in mice with DIO. A combined diet-periodontal ligation model was established. The weight of mice induced by an HF diet for 15–16 weeks, which exceeded 10 g or 20–25% higher than that of control mice, was generally taken as obesity standard for rodents [26]. The C57BL/6J mouse strain, seemingly susceptible to the development of metabolic syndrome, must consume an HF diet and remain obese for a long duration before the pulmonary phenotype characteristics of obese mice are observed [27]. In this regard, we combined mouse models of DIO and ligation-induced periodontitis by feeding a 60% kcal HF diet for 30 weeks plus 10 days and observed that HF group had 50% body weight gain compared with that of LF group. It is also acknowledged that obesity reflects a state of low-grade systemic inflammation and immune dysfunction, with inflammatory activation at sites distant to the adipose tissue. Previous studies demonstrated that obese mouse models exhibited sterile lung injury and inflammation, such as innate airway hyper-responsiveness and macrophage recruitment [28,29]. Similarly, we found that obese mice induced by HF were characterized by alveolar septum thickening and bronchial secretions, which were more evident than LF mice, as well as elevated macrophage marker F4/80 protein and MCP1 levels in lung tissue. These findings suggest that recruitment of alveolar macrophages to lung focus was enhanced in the context of obesity, but the regulation caused by periodontitis was not significant. Compared with high diet mice with no periodontitis, DIO mice with periodontitis tended to downregulate alveolar macrophage recruitment. Several studies demonstrated that obese mice had an ameliorative or augmentative effect on lung injury and inflammation after *Escherichia coli* LPS [30], hyperoxia [31], ozone [20], or particulate exposures [21]. The inconsistent results are due in large part to the type and degree of initiating injury and the timing of injury and examination [32]. TNF-α, IL-1β, IL-6, and IL-10 are early-response mediators, which are largely secreted by innate immune cells, including macrophages [33]. In this study, TNF-α, IL-1β, and IL-6 levels were upregulated in the lung of DIO mice; however, IL-10 level was unaffected by either periodontitis or obesity, which implicates the imbalance of the pro- and anti-inflammatory cytokine protein status of the lung lesion in obese mice coupled with periodontitis. We also observed that periodontitis induced by ligation with *P. gingivalis* can dramatically upregulate the TNF-α level in lung tissue under LF diet. However, DIO mice with periodontitis showed sluggish inflammatory response with reduced expression of TNF-α level compared with that of DIO mice. Furthermore, TNF-α and IL-6 were more likely to be downregulated in DIO mice with periodontitis when compared with their obesity-only counterparts. Similar behavior was identified in DIO mice with altered immune responses when infected with *P. gingivalis*. Specifically, the animals with DIO for 16 weeks and subjected to oral infection or systemic inoculation of live *P. gingivalis* showed a blunted inflammatory response with reduced expression of serum TNF-α and IL-6 compared with that of lean mice [34]. Some reports evidenced that periodontal infection in the context of obesity dramatically affected the regional and systemic immune systems. Interestingly, Zuomin Wang [14] reported that, relative to that of the control group, the mRNA expression level of cytokines TNF-*α* in the lung tissue of the ligature plus *P. gingivalis*-induced periodontitis groups increased significantly at 8 weeks but otherwise not obviously at 2 weeks. Our result showed that periodontitis induced by ligation with *P. gingivalis* could upregulate TNF-α levels in lung tissue under an LF diet at 10 days. The different findings can be attributed to the different settings of the control group. Specifically, our study used an LF diet as control, upon which the mice gained weight of 12 g compared to that of the initial stage. In other words, mice under an LF diet for 30 weeks plus 10 days were also obese compared to their initial states. It was quite different from the control group used in Zuomin Wang’s study, in which the mice were fed a normal diet for 2 weeks. This finding implies that obese treatment for a long duration may exacerbate lung changes when subjected to periodontitis.

To the best of our knowledge, it is the first report to demonstrate that periodontitis affects the lung inflammation state of DIO animal models. Despite the encouraging findings, the limitations are also included based on experimental results. First, ligation for 10 days seemingly induces a subacute form of periodontal destruction in mice, thus distinguishing it from the chronic lesion of periodontitis in the human body. However, the early-stage symptoms or acute status of periodontal diseases may also occur. Obesity triggered by an HF diet would be more complicated, upon which the individual effects of metabolic syndrome elements on pulmonary injury lesion, such as hyperglycemia, dyslipidemia, hypoadiponectinemia, and hyperleptinemia, are difficult to be unambiguously identified. Second, the underlying mechanism behind the fact that periodontitis influences pulmonary immune status in obese mice remains to be fully unveiled. Further endeavors are necessary to elucidate cytological and molecular effects on pulmonary immune dysregulation and systemic events both *in vitro* and *in vivo*.

## 5 Conclusion

In conclusion, we presented evidence that periodontitis influences the innate immune response of pulmonary in the context of obesity. This is presumably related to the imbalance of the pro- and anti-inflammatory cytokine protein status of the lung lesion, which tended to attenuate infiltration of alveolar macrophages. This study highlights the importance of prophylaxis and treatment of periodontitis in obese individuals with a respiratory disorder.

## References

[1] Slots J. Periodontitis: facts, fallacies and the future. Periodontol 2000. 2017;75(1):7–23. 10.1111/prd.12221 28758294

[2] GBD 2017 Oral Disorders Collaborators, Bernabe E, Marcenes W, Hernandez CR, Bailey J, Abreu LG, Alipour V, et al. Global, regional, and national levels and trends in burden of oral conditions from 1990 to 2017: a systematic analysis for the global burden of disease 2017 study. J Dent Res. 2020;99(4):362–73. 10.1177/0022034520908533 PMC7088322 32122215

[3] Ahmad FB, Anderson RN. The leading causes of death in the US for 2020. JAMA. 2021 May 11;325(18):1829–30. 10.1001/jama.2021.5469 PMC8145781 33787821

[4] Genco RJ, Sanz M. Clinical and public health implications of periodontal and systemic diseases: An overview. Periodontol 2000. 2020 Jun;83(1):7–13. 10.1111/prd.12344 32385880

[5] Marouf N, Cai W, Said KN, Daas H, Diab H, Chinta VR, et al. Association between periodontitis and severity of COVID-19 infection: A case-control study. J Clin Periodontol. 2021 Apr;48(4):483–91. 10.1111/jcpe.13435 PMC8014679 33527378

[6] Romandini M, Baima G, Antonoglou G, Bueno J, Figuero E, Sanz M. Periodontitis, edentulism, and risk of mortality: A systematic review with meta-analyses. J Dent Res. 2021 Jan;100(1):37–49. 10.1177/0022034520952401 32866427

[7] Gomes-Filho IS, Cruz SSD, Trindade SC, Passos-Soares JS, Carvalho-Filho PC, Figueiredo ACMG, et al. Periodontitis and respiratory diseases: A systematic review with meta-analysis. Oral Dis. 2020;26(2):439–46. 10.1111/odi.13228 31715080

[8] Lopes MP, Cruz ÁA, Xavier MT, Stöcker A, Carvalho-Filho P, Miranda PM, et al. Prevotella intermedia and periodontitis are associated with severe asthma. J Petiodontol. 2020;91(1):46–54. 10.1002/JPER.19-0065 31342509

[9] Mammen MJ, Scannapieco FA, Sethi S. Oral-lung microbiome interactions in lung diseases. Periodontol 2000. 2020 Jun;83(1):234–41. 10.1111/prd.12301 32385873

[10] He Y, Shiotsu N, Uchida-Fukuhara Y, Guo J, Weng Y, Ikegame M, et al. Porphyromonas gingivalis induced cell death with disruption of tight junctions in human lung epithelial cells. Arch Oral Bio. 2020;118:104841. 10.1016/j.archoralbio.2020.104841 32717445

[11] Mei F, Xiu M, Huang X, Long Y, Lu X, Wang X, et al. Porphyromonas gingivalis and its systemic impact: current status. Pathogens. 2020;9(11):944. 10.3390/pathogens9110944 PMC7696708 33202751

[12] Watanabe N, Yokoe S, Ogata Y, Sato S, Imai K. Exposure to Porphyromonas gingivalis induces production of proinflammatory cytokine via TLR2 from human respiratory epithelial cells. J Clin Med. 2020;9(11):3433. 10.3390/jcm9113433 PMC7693763 33114582

[13] Hamamoto Y, Ouhara K, Munenaga S, Shoji M, Ozawa T, Hisatsune J, et al. Effect of Porphyromonas gingivalis infection on gut dysbiosis and resultant arthritis exacerbation in mouse model. Arthritis Res Ther. 2020 Oct 19;22(1):249. 10.1186/s13075-020-02348-z PMC7574451 33076980

[14] Tian H, Zhang Z, Wang X, Liu W, Wang Z. Role of experimental periodontitis in inducing pulmonary inflammation in mice [published online ahead of print, 2021 Jun 26]. Oral Dis. 2021;10.1111/odi.13949. 10.1111/odi.13949 34174133

[15] Suvan JE, Finer N, D’Aiuto F. Periodontal complications with obesity. Periodontol 2000. 2018 Oct;78(1):98–128. 10.1111/prd.12239 30198136

[16] Memtsoudis SG, Ivascu NS, Pryor KO, Goldstein PA. Obesity as a risk factor for poor outcome in COVID-19-induced lung injury: the potential role of undiagnosed obstructive sleep apnoea. Br J Anaesth. 2020;152(2):e262–3. 10.1016/j.bja.2020.04.078 PMC7252174 32439072

[17] Popkin BM, Du S, Green WD, Beck MA, Algaith T, Herbst CH, et al. Individuals with obesity and COVID-19: A global perspective on the epidemiology and biological relationships. Obes Rev. 2020 Nov;21(11):e13128. 10.1111/obr.13128 PMC7461480 32845580

[18] Mouton AJ, Li X, Hall ME, Hall JE. Obesity, hypertension, and cardiac dysfunction: novel roles of immunometabolism in macrophage activation and inflammation. Cir Res. 2020;126:789–806. 10.1161/CIRCRESAHA.119.312321 PMC7255054 32163341

[19] Park YH, Oh EY, Han H, Yang M, Park HJ, Park KH, et al. Insulin resistance mediates high-fat diet-induced pulmonary fibrosis and airway hyperresponsiveness through the TGF-β1 pathway. Exp Mol Med. 2019;51:1–12. 10.1038/s12276-019-0258-7 PMC6536500 31133649

[20] Tashiro H, Cho Y, Kasahara DI, Brand JD, Bry L, Yeliseyev V, et al. Microbiota contribute to obesity-related increases in the pulmonary response to ozone. Am J Respir Cell Mol Biol. 2019;61(6):702–12. 10.1165/rcmb.2019-0144OC PMC6890400 31144984

[21] Leikauf GD, Kim SH, Jang AS. Mechanisms of ultrafine particle-induced respiratory health effects. Exp Mol Med. 2020;52:329–37. 10.1038/s12276-020-0394-0 PMC7156674 32203100

[22] Kimura S, Nagai A, Onitsuka T, Koga T, Fujiwara T, Kaya H, et al. Induction of experimental periodontitis in mice with Porphyromonas gingivalis-adhered ligatures. J Periodontol. 2000 Jul;71(7):1167–73. 10.1902/jop.2000.71.7.1167 10960025

[23] Li CH, Amar S. Morphometric, histomorphometric, and microcomputed tomographic analysis of periodontal inflammatory lesions in a murine model. J Periodontol. 2007;78(6):1120–8. 10.1902/jop.2007.060320 17539727

[24] National Institutes of Health. Guide for the Care and Use of Laboratory animals. 8th edn. Washington: National Academies Press, US; 2011.

[25] Yu T, Zhao L, Huang X, Xie M, Wang X, Ma C, et al. Postoperative weight loss masks metabolic impacts of periodontitis in obese rodents. J Periodontol. 2017;88(6):e97–e108. 10.1902/jop.2017.160655 28394188

[26] Wang P, Li D, Ke W, Liang D, Hu X, Chen F. Resveratrol-induced gut microbiota reduces obesity in high-fat diet-fed mice. Int J Obes. 2020;44:213–25. 10.1038/s41366-019-0332-1 30718820

[27] Avtanski D, Pavlov VA, Tracey KJ, Poretsky L. Characterization of inflammation and insulin resistance in high-fat diet-induced male C57BL/6J mouse model of obesity. Anim Model Exp Med. 2019;2(4):252–8. 10.1002/ame2.12084 PMC6930989 31942557

[28] Huang WC, Liu CY, Shen SC, Chen LC, Yeh KW, Liu SH, et al. Protective effects of Licochalcone A improve airway hyper-responsiveness and oxidative stress in a mouse model of asthma. Cells. 2019;8(6):617. 10.3390/cells8060617 PMC6628120 31226782

[29] Russo L, Lumeng CN. Properties and functions of adipose tissue macrophages in obesity. Immunology. 2018;155(4):407–17. 10.1111/imm.13002 PMC6230999 30229891

[30] Guo H, Zuo Z, Wang F, Gao C, Chen K, Fang J, et al. Attenuated Cardiac oxidative stress, inflammation and apoptosis in Obese Mice with nonfatal infection of Escherichia coli. Ecotoxi Env Saf. 2021;225:112760. 10.1016/j.ecoenv.2021.112760 34509165

[31] Zheng G, Ren H, Li H, Li X, Dong T, Xu S, et al. Lycium barbarum polysaccharide reduces hyperoxic acute lung injury in mice through Nrf2 pathway. Biom Pharma. 2019;111:733–9. 10.1016/j.biopha.2018.12.073 30611998

[32] Suratt BT. Mouse modeling of obese lung disease. insights and caveats. Am J Respir Cell Mol Biol. 2016;55(2):153–8. 10.1165/rcmb.2016-0063PS PMC4979373 27163945

[33] Shapouri-Moghaddam A, Mohammadian S, Vazini H, Taghadosi M, Esmaeili SA, Mardani F, et al. Macrophage plasticity, polarization, and function in health and disease. J Cell Physi. 2018;223(9):6425–40. 10.1002/jcp.26429 PMC13482560 29319160

[34] Amar S, Zhou Q, Shaik-Dasthagirisaheb Y, Leeman S. Diet-induced obesity in mice causes changes in immune responses and bone loss manifested by bacterial challenge. Proc Natl Acad Sci U S A. 2007;104(51):20466–71. 10.1073/pnas.0710335105 PMC2154454 18077329

